# Effects of stand age and soil properties on soil bacterial and fungal community composition in Chinese pine plantations on the Loess Plateau

**DOI:** 10.1371/journal.pone.0186501

**Published:** 2017-10-19

**Authors:** Peng Dang, Xuan Yu, Hien Le, Jinliang Liu, Zhen Shen, Zhong Zhao

**Affiliations:** 1 College of Forestry, Northwest A&F University, Yangling, China; 2 State Key Laboratory of Soil Erosion and Dryland Farming on the Loess Plateau, Northwest A&F University, Yangling, China; Friedrich Schiller University, GERMANY

## Abstract

The effects of Chinese pine (*Pinus tabuliformis*) on soil variables after afforestation have been established, but microbial community changes still need to be explored. Using high-throughput sequencing technology, we analyzed bacterial and fungal community composition and diversity in soils from three stands of different-aged, designated 12-year-old (PF1), 29-year-old (PF2), and 53-year-old (PF3), on a Chinese pine plantation and from a natural secondary forest (NSF) stand that was almost 80 years old. Abandoned farmland (BL) was also analyzed. Shannon index values of both bacterial and fungal community in PF1 were greater than those in PF2, PF3 and NSF. *Proteobacteria* had the lowest abundance in BL, and the abundance increased with stand age. The abundance of *Actinobacteria* was greater in BL and PF1 soils than those in other sites. Among fungal communities, the dominant taxa were *Ascomycota* in BL and PF1 and *Basidiomycota* in PF2, PF3 and NSF, which reflected the successional patterns of fungal communities during the development of Chinese pine plantations. Therefore, the diversity and dominant taxa of soil microbial community in stands 12 and 29 years of age appear to have undergone significant changes; afterward, the soil microbial community achieved a relatively stable state. Furthermore, the abundances of the most dominant bacterial and fungal communities correlated significantly with organic C, total N, C:N, available N, and available P, indicating the dependence of these microbes on soil nutrients. Overall, our findings suggest that the large changes in the soil microbial community structure of Chinese pine plantation forests may be attributed to the phyla present (e.g., *Proteobacteria*, *Actinobacteria*, *Ascomycota* and *Basidiomycota*) which were affected by soil carbon and nutrients in the Loess Plateau.

## Introduction

Soil bacterial and fungal communities play important roles in the decomposition of organic matter and provide available nutrients for plant growth [1, 2]. As such, these communities are dynamic components of terrestrial ecosystems and exhibit temporal and spatial variation [3, 4]. Previous studies have shown that vegetation changes with changes in land use [5, 6]. These land use changes directly affect litter, root and exudates that indirectly affect soil organic carbon (SOC) and other edaphic properties of the soil that eventually translate into alterations in ecological succession [7, 8]. Microbial communities are influenced by pH, SOC, and other soil factors [9, 10]. Additionally, soil organisms, nutrients and associated microbial communities are influenced by changes in land use or forest growth [5].

Microbial community structure and activity change during ecological restoration of abandoned agricultural soils [11, 12] and post-mining areas [13, 14] through plant secondary succession [15, 16]. Belowground properties, including plant diversity and richness, soil pH, total carbon, total nitrogen and other nutrient content, change simultaneously with changes in the aboveground structure. Furthermore, these indexes ultimately influence the structure and function of the soil microbial community [17–19]. In forest ecosystems, afforestation may alter the physicochemical and activity properties of the soil due to changes in the forest tree structure [20, 21]. Determining the soil microbial community dynamics that reflect changes in forest age and soil properties is important when evaluating the interplay between aboveground and belowground communities.

The Loess Plateau has an area of approximately 9.6×10^6^ ha and lies in the upper-middle reaches of the Yellow River in China [22]. This region experiences serious soil erosion and drought. To accelerate ecological rehabilitation and improve ecological stability in this region, a project called “Grain for Green” has been implemented on the Loess Plateau. This project aims to improve the fragile ecosystem by converting farmland into forestland [23]. As the predominant pioneer tree species, and Chinese pine is widely planted for Loess Plateau afforestation due to its high tolerance to cold, drought, and poor soil quality [24, 25]. Previous studies of Chinese pine forests have focused primarily on the aboveground ecosystem, soil chemistry, enzyme activity and microorganisms across different stand ages or management strategies [26]. For example, a PCR-DGGE approach was used to characterize soil bacterial and fungal communities in the rhizosphere of 30-year-old Chinese pine stands on the Loess Plateau [27]. However, successional changes in soil bacterial and fungal communities, which are crucial for the assessment of ecological restoration in Chinese pine plantations, have never been studied.

The study aimed (1) to examine the bacterial and fungal community structure composition and diversity of a Chinese pine plantation in stands of different ages and (2) to reveal the relationship between soil physicochemical properties and microbial communities. We employed sequencing technology to identify and study microbial groups with relatively low abundance (<1%) [28], quantify and model successional dynamics, such as those associated with microbial community structure and diversity, and characterize relationship between microorganisms and soils [29, 30].

## Materials and methods

### Study area description

**Fig 1:**
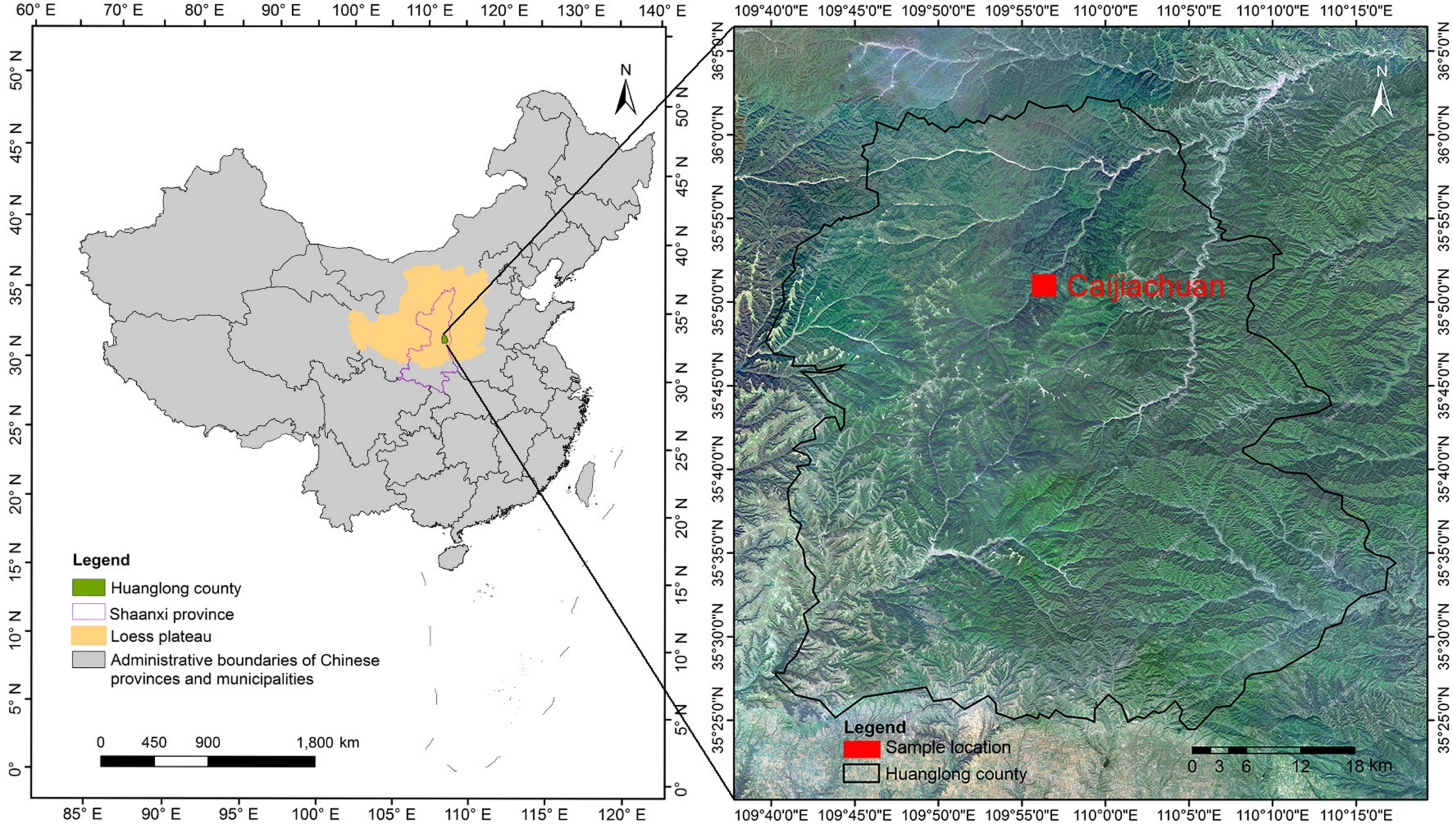
Map of the study sites located in the Caijiachuan forest area of Huanglong county on the Loess Plateau.

The study was conducted in the Chinese pine forests located in the Caijiachuan forest area of Huanglong County (35°28′–36°02′N, 109°38′–110°12′E), which is on the southern Loess Plateau in northwestern China (Fig 1). Permission was granted by the Huanglong Forestry Bureau to carry out our research. The area of the Loess Plateau is characterized by many hills and gullies, with an altitude of 1200 meters. The study area is in the transition zone between semi-humid and semi-arid. Its climate is warm and temperate with a mean annual temperature of 8.6°C. The annual precipitation is 611.8 mm, with the majority of rainfall falling between July and September. The frost-free season averages 158 days in length. The Loess Plateau receives an average of 2369.8 h of daylight annually. The soil type is Typic-Loessi Orthic Primosols [31].

Large areas of abandoned farmlands with low yields were converted into forest land to improve the fragile ecosystem on the Loess Plateau decades ago. As the dominant native tree species, Chinese pine was planted beginning in the 1960s. Before planting, the main crop species were maize (*Zea mays subsp*), millet (*Setaria italica*) and pea (*Pisum sativum Linn*), and the only moisture applied during the growing seasons was derived from the falling rain. In this study, we collected samples from three stands of different ages that were planted in 1962, 1986 and 2003. For comparison with the plantation samples, control samples were obtained from farmland left uncultured for three years and an 80-year-olds Chinese pine natural secondary forest.

### Sampling

Sampling was conducted in August 2015. Stands of the following three ages from the Chinese pine plantation were examined: 12-years (PF1), 29-years (PF2) and 53-years (PF3). One abandoned farmland site and one natural secondary Chinese pine forest stand with an age of almost 80 years (NSF) were selected as controls. The basic characteristics of the different stand sites in the Chinese pine forest and abandoned farmland are described in Table 1.

**Table 1:** The basic conditions of the forests and abandoned farmland sites.

Stand sites	Age (year)	Mean DBH (m)	Canopy closure (%)	Shrubs Coverage (%)	Herbs Coverage (%)	Slope aspect	Gradient	Altitude (m)
BL	-	-	-	-	55.2	260°	16.5°	1296
PF1	15	6.3	52.5	10.2	62.5	275°	15.0°	1430
PF2	29	11.8	82.2	35.6	28.8	235°	12.5°	1499
PF3	55	21.2	78.6	40.3	22.2	325°	18.0°	1321
NSF	80	29.4	70.8	32.5	18.3	265°	20.5°	1283

BL, PF1, PF2, PF3 and NSF represent abandoned land, 12-year-old plantation forest, 29-year-old forest, 53-year-old plantations forest and NSF with an age of almost 80 years, respectively.

Three replicate plots (20 m × 20 m) with similar slopes, gradients and altitudes were established at each stand site. The distance between each sampling plot was approximately 100 m. For each triplicate plot, nine soil corns of topsoil (0~10 cm) were collected with an “S” shape after removing the litter layer and then mixed to form one sample. A stainless-steel corer with a 4.5-cm inner diameter was used to collect soil. Fifteen samples were collected in total (five stand sites × three plots). All samples were filtered to remove the roots, litter and stones and then transported to the laboratory on ice immediately. One aliquot of each sample was stored at -80°C for DNA extraction. The remaining aliquots were air-dried and used to analyze physicochemical properties.

### Analyses of soil physicochemical properties

The soil pH was determined using an electrode pH meter (Sartorius PB-10, Germany) after shaking the soil water liquid suspension (1:5 wt/vol) [32]. The soil total carbon (SOC) was determined using an Elemental analyzer (Elementar, Germany). Total nitrogen (TN) content was assessed via the Kjeldahl method [33]. Ammonium nitrogen (NH_4_^+^-N) and nitrate nitrogen (NO_3_^-^-N) levels were measured using an AA3 continuous flow analytical system with 1 M KCl extraction (AA3, Germany) [32]. Total phosphorus (TP) was measured by spectrophotometry after wet digestion with HClO_4_-H_2_SO_4_ [34]. Available phosphorus (AP) was measured using the colorimetric method with 0.5 M NaHCO_3_ extraction [35].

### Soil DNA extraction

E.Z.N.A soil DNA kits (OMEGA, USA) were used to extract total soil microbial community DNA in triplicate per soil sample. Briefly, 0.5 g of soil was placed into tubes and processed with buffer containing detergent. Then, a heating-and-freezing process was conducted to precipitate soil contaminants such as protein and humic acid. Next, water was used to elute the pure DNA. Subsequently, the purified DNA was quantified using a spectrophotometer (Epoch, BioTek, USA).

### PCR amplification and sequencing of 16S rRNA and 18S rRNA genes

The V3-V4 region of bacterial 16S rDNA genes was amplified by PCR using primers 338F (5’-barcode-ACTCCTACGGGAGGCAGCAG)-3’) and 806R (5’-GGACTACHVGGGTWTCTAAT-3’) [36]. The following PCR procedure was used: 95°C for 3min, followed by 27 cycles of 95°C for 30 s, 55°C for 30 s, and 72°C for 45 s with a final extension at 72°C for 10 min. The PCR reactions were performed in a 20 μL reaction volume (2 μL of 2.5 mM dNTPs, 4 μL of 5× FastPfu Buffer, 0.4 μL of FastPfu Polymerase, 0.8 μL of each primer at 5 μM, and 10 ng of template DNA). The primers for the fungal 18S rDNA genes were SSU0817F (5’-barcode-TTAGCATGGAATAATRRAATAGGA)-3’) and SSU1196R (5’-TCTGGACCTGGTGAGTTTCC-3’) [37]. The following PCR procedure was used: 95°C for 3 min, followed by 32 cycles of 95°C for 30 s, 55°C for 30 s, and 72°C for 45 s with a final extension at 72°C for 10 min. The PCR reactions were performed in a 20 μL reaction volume (4 μL of 5× FastPfu Buffer, 2 μL of 2.5 mM dNTPs, 0.4 μL of FastPfu Polymerase, 0.8 μL of each primer at 5 μM primer, and 10 ng of template DNA). Triplicate amplicons were extracted from 2% agarose gels and purified using a DNA Extraction Kit. Then, the DNA amplicons were combined in an equimolar ratio and analyzed via sequencing using the Illumina MiSeq platform.

### Processing of sequencing data

Raw fastq files were demultiplexed and quality filtered using QIIME (version 1.17) as follows [38]. First, the 300 bp reads were truncated at any site that received an average quality score <20 over a 50 bp sliding window. Second, reads containing ambiguous characters and exact barcode matches were removed. Third, only sequences that had an overlap longer than 10 bp were assembled according to their overlapping sequence. Reads that could not be assembled were discarded. UPARSE (version 7.1 http://drive5.com/uparse/) was used to cluster the operational taxonomic units (OTUs) using a 97% similarity cutoff [39]. UCHIME was used to identify and remove chimeric sequences [40].

The taxonomy of each 16S rRNA gene sequence was analyzed with the RDP classifier (http://rdp.cme.msu.edu/) against the Silva reference database (http://www.arb-silva.de) using a confidence threshold of 70% (version 2013). The phylogenetic affiliation of each 18S rRNA gene sequence was analyzed with the RDP classifier (http://rdp.cme.msu.edu/) against the Silva (SSU117/119) 18S rRNA database using a confidence threshold of 70% [41].

### Statistical analyses

All soil environmental factors (SOC, TN, TP, AP, NH_4_^+^-N, NO_3_^-^-N and pH), microbial community diversity indexes (Shannon and Simpson), and the relative abundances of microbial communities were compared by performing one-way analysis (ANOVA). Multiple comparisons were carried out to compare the differences among sample sites according to the least significant difference (LSD). Principal coordinates analysis (PCoA) was used to assess the microbial community structures in the stands based on Bray-Curtis distances. Redundancy analysis (RDA) was applied to test the relationships between soil physicochemical properties and the major microbial groups. Analysis of variance was performed using SPSS version 11.5 software (SPSS Inc., Chicago, IL, USA). PCoA and RDA were performed using the R software package (V.3.4.0).

## Results

### Soil physicochemical properties

The SOC and TN concentrations and the C:N ratios in all forests samples increased significantly compared to those in the abandoned land samples(*P*<0.01). The concentrations of NO_3_^-^-N in PF3 and NSF were significantly higher than those in BL, PF1 and PF2 (*P*<0.05). AP concentration and soil bulk density (BD) decreased with plantation age (Table 2).

**Table 2:** Soil physicochemical characteristics of forest and abandoned land of different ages.

	BL	PF1	PF2	PF3	NSF
pH	8.23±0.03	8.20±0.04	8.21±0.07	8.20±0.03	8.19±0.02
SOC(g/kg)	12.83±0.75c	17.97±1.40b	20.33±2.80ab	20.00±1.31ab	21.90±0.80a
TN(g/kg)	1.02±0.03b	1.20±0.02a	1.29±0.08a	1.23±0.09a	1.27±0.045a
TP(g/kg)	0.58±0.01	0.56±0.04	0.60±0.08	0.57±0.01	0.56±0.01
C:N	12.58±0.62c	15.02±0.63b	15.63±1.20ab	16.29±1.10ab	17.21±0.57a
NH_4_^+^-N(mg/kg)	3.35±0.18b	3.90±0.11b	3.68±0.68b	3.94±0.61b	5.14±0.46a
NO_3_^-^-N(mg/kg)	1.13±0.12b	1.30±0.08b	1.17±0.06b	1.60±0.16a	1.72±0.43a
AP(mg/kg)	11.28±0.21a	9.73±1.21b	9.51±0.13b	9.15±0.61b	8.81±2.56b
SWC(%)	10.27%±0.12%	11.26%±0.39%	10.84±0.73%	11.26%±0.55%	11.03%±0.16%
BD(g/cm^3^)	1.18±0.06a	1.14±0.06a	1.12±0.10a	0.83±0.05b	0.94±0.08b

Lowercase letters indicate significant differences (*P*<0.05) among the five sample stands. SOC, soil organic carbon; TN, soil total nitrogen; TP, soil total phosphorus; C: N, soil organic carbon and total nitrogen ratio; NH_4_^+^-N, ammonium nitrogen; NO_3_^-^-N, nitrate nitrogen; AP, available phosphorus; SWC, soil water content; BD, soil bulk density.

### Alpha and beta diversity of bacterial and fungal communities

**Fig 2:**
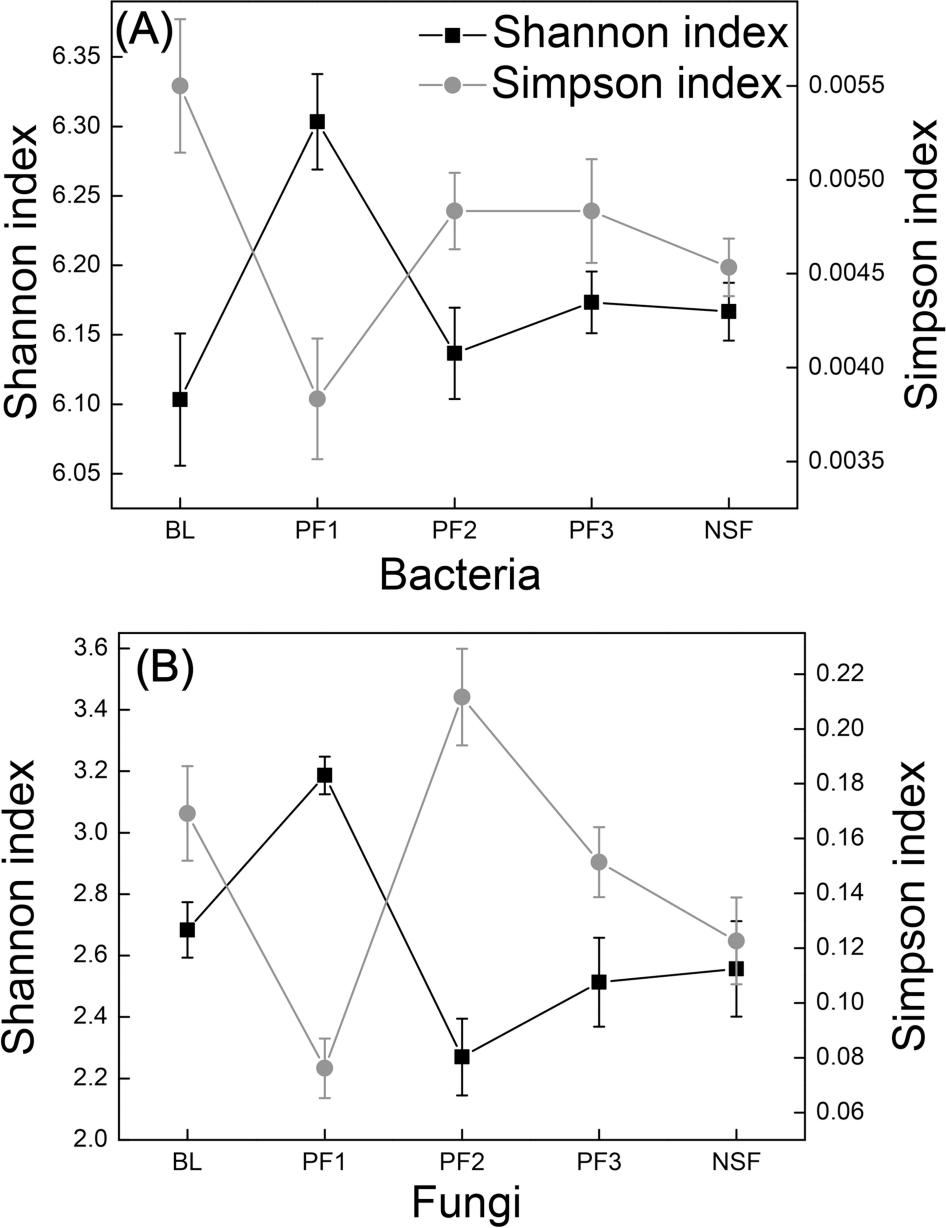
Shannon index and Simpson index of bacterial (a) and fungal (b) communities in plantation stands of different ages and abandoned farmland.

The dataset comprised a total of 332,172 high-quality bacterial sequences after the elimination of chimeras, with an average of 22,145 sequences obtained from each soil sample. A total of 20,661 OTUs were identified at a 97% sequence similarity cutoff, with an average of 1,377 OTUs per sample. The rarefaction curves of all the soil samples are shown in S1A Fig. The Shannon index of PF1 was significantly higher than that of PF2, PF3, and NSF. However, the Simpson index was lower in PF1 than that in PF2, PF3, and NSF (Fig 2A).

After the elimination of chimeras, a total of 477,045 fungal sequences were obtained from all soil samples. A total of 1,792 OTUs were identified at a 97% sequence similarity cutoff, with an average of 119 OTUs per sample. The rarefaction curves of all of the soil samples are shown in S1B Fig. The Shannon indexes of BL and PF1 were significantly higher than those of PF2, PF3, and NSF (*P*<0.05). However, the Simpson indexes of BL and PF1 were significantly lower than those of PF3 and NSF, respectively (*P*<0.05) (Fig 2B).

**Fig 3:**
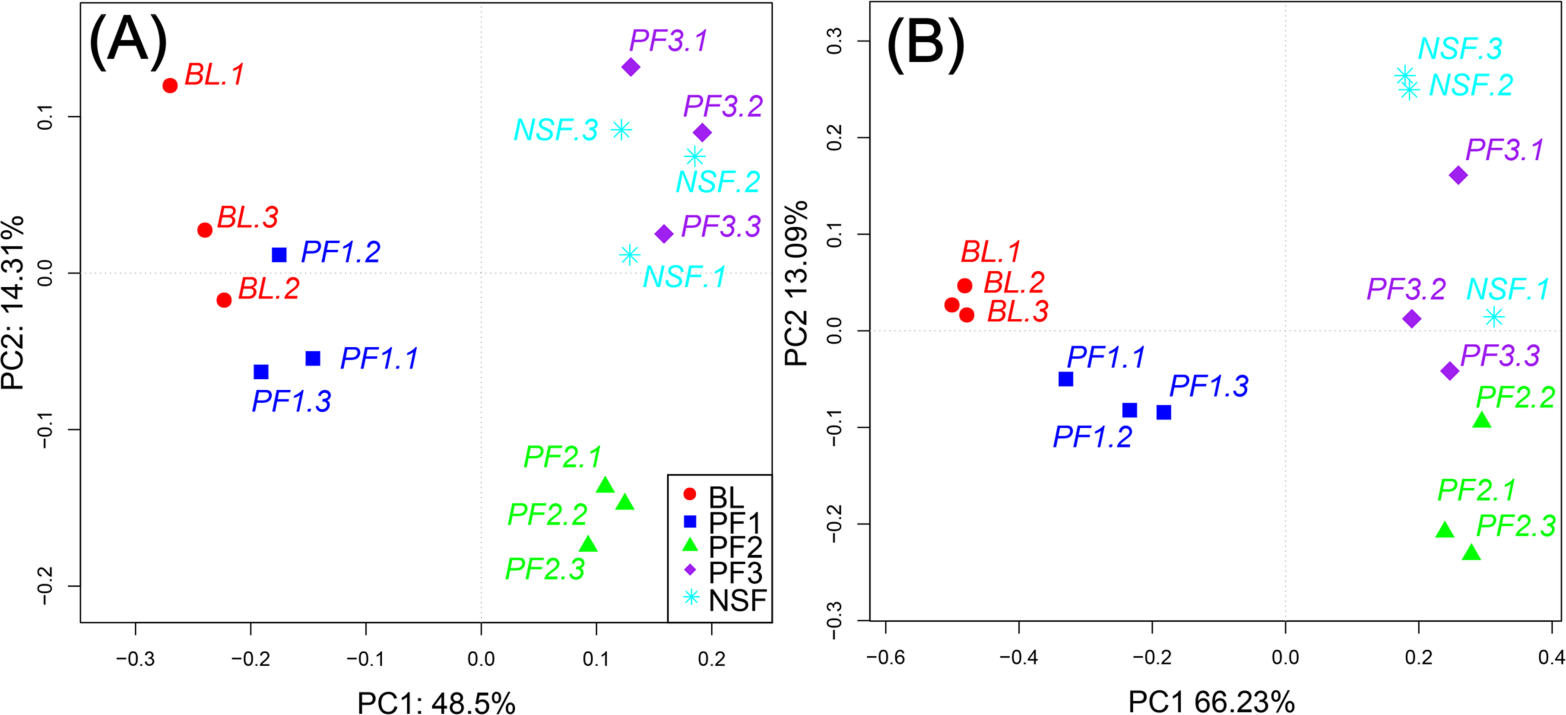
PCoA for soil bacterial (a) and fungal (b) communities from forest sites (PF1, PF2, PF3, NSF) and abandoned land (BL).

PCoA revealed a variation in the bacterial and fungal communities among all soil sample sites (Fig 3). For all the groups of bacterial communities, the first and second principal components explained 48.5% and 13.21% of the variance, respectively (Fig 3A). For fungal communities, the first and second principal components explained 66.23% and 13.09% of the variance, respectively (Fig 3B). Profiles of soil bacterial communities from the BL and PF1 sites tended to group together, while the PF3 and NSF sites also tended to group together. The fungal communities showed a trend similar to that of the bacterial communities.

### Composition of the bacterial and fungal communities

**Fig 4:**
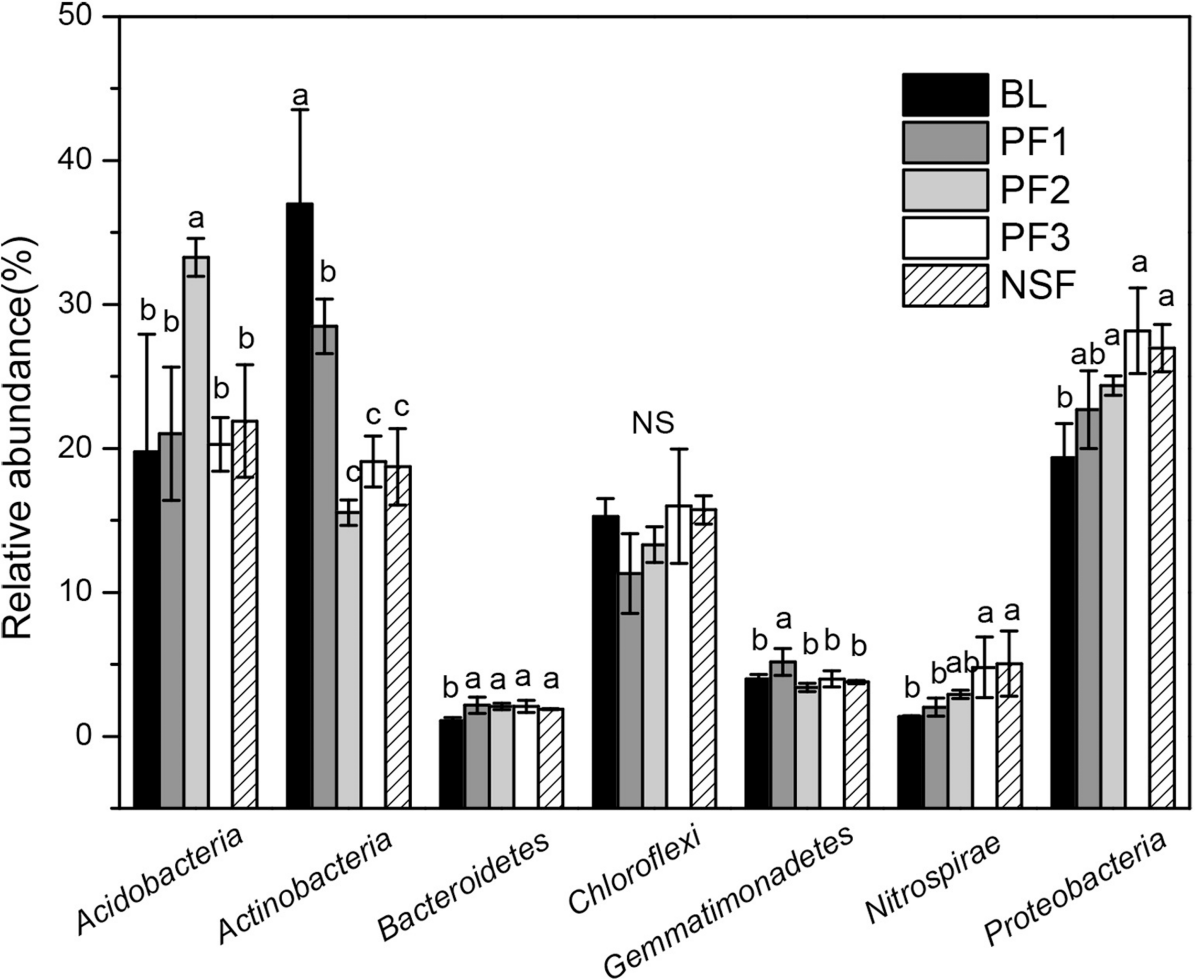
Mean relative abundances of dominant bacterial phyla across all different sites. Different letters indicate signiFIcant differences (P<0.05) across the different sample sites. NS: not significant.

The dominant bacterial phyla were *Proteobacteria* (25.11%), *Actinobacteria* (23.77%), *Acidobacteria* (23.25%), *Chloroflexi* (14.32%), *Gemmatimonadetes* (4.06%), *Nitrospirae* (3.23%), *Bacteroidetes* (1.86%) and *Planctomycetes* (1.06%) (Fig 4). The relative abundances of the *Proteobacteria* subgroups (*Alpha-*, *Beta-*, *Gamma-*, and *Delta-Proteobacteria*) were 12.37%, 6.98%, 2.14%, and 4.14%, respectively (S2 Fig). The *Alpha-proteobacteria* sequences were dominated by *Rhizobiales* (6.87%), *Sphingomonadales* (2.40%), and *Rhodospirillales* (2.38%). MND1 (3.21%), *Xanthomonadales* (1.57%), and *Syntrophobacterales* (1.70%) were the most abundant groups within the *Beta-proteobacteria*, *Gamma-proteobacteria*, and *Delta-proteobacteria*, respectively (S3 Fig). The relative abundances of *Proteobacteria* and *Bacteroidetes* were significantly lower in the BL site than those in all other forest sites. *Rhizobiales*, *Rhodospirillales*, and *Xanthomonadales* all had the same variance. The relative abundance of *Actinobacteria* were significantly higher in the BL and PF1 sites than those in the older stands (PF2, PF3 and NSF) (Fig 4 and S4 Fig). The relative abundances of *Nitrospirae* in the NSF (5.05%) and PF3 (4.79%) sites were significantly higher than those in the PF2 (2.92%), PF1 (2.02%), and BL (1.38%) sites (Fig 4).

**Fig 5:**
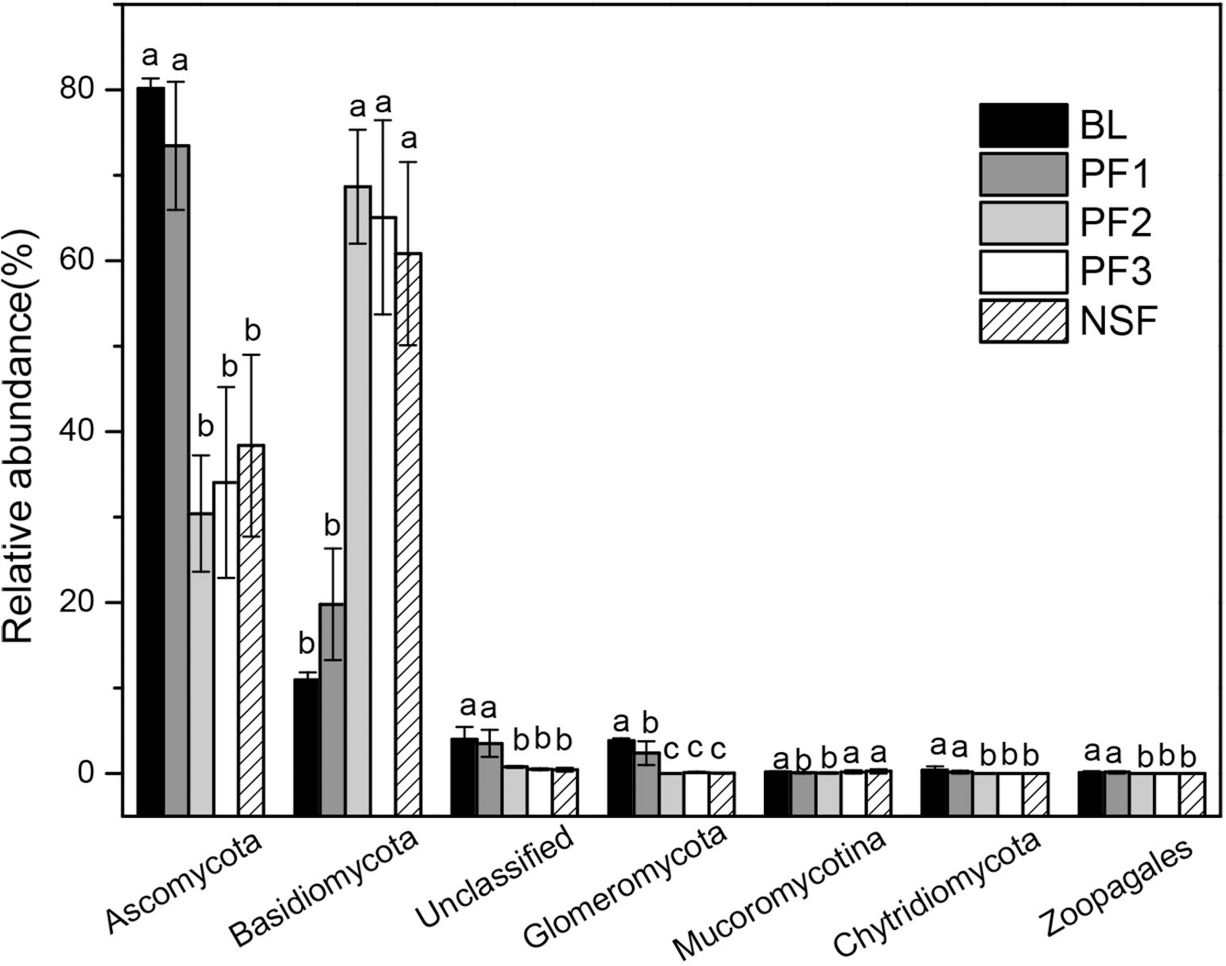
Mean relative abundances of dominant fungal phyla across all different sites. Different letters indicate significant differences (P<0.05) across the different sample sites.

*Ascomycota* and *Basidiomycota* sequences were the most abundant among the fungal communities in all soil samples, representing 51.29% and 45.06% of all sequences, respectively (Fig 5). The relative abundances of *Ascomycota* were significantly higher in the BL (80.19%) and PF1 (73.43%) sites than those in the PF2 (30.40%), PF3 (34.04%), and NSF (38.38%) sites. The relative abundances of *Basidiomycota* were significantly lower in the BL (10.94%) and PF1 (19.79%) sites than those in the PF2 (68.67%), PF3 (65.07%), and NSF (60.83%) sites. The remaining taxa included *Glomeromycota* (≈1.2%), *Mucoromycotina* (<1%), *Chytridiomycota* (<1%), and *Zoopagales* (<1%) (Fig 5). Of the *Ascomycota*, *Sordariomycetes* were the most abundant (16.58%), followed by *Pezizomycetes* (13.86%), *Dothideomycetes* (8.07%) and *Eurotiomycetes* (5.93%). Of the *Basidiomycota*, *Agaricomycetes* (42.21%) and *Tremellomycetes* (2.49%) were the most abundant groups (S5 Fig).

### Correlation between microbial communities and soil physicochemical properties

**Fig 6:**
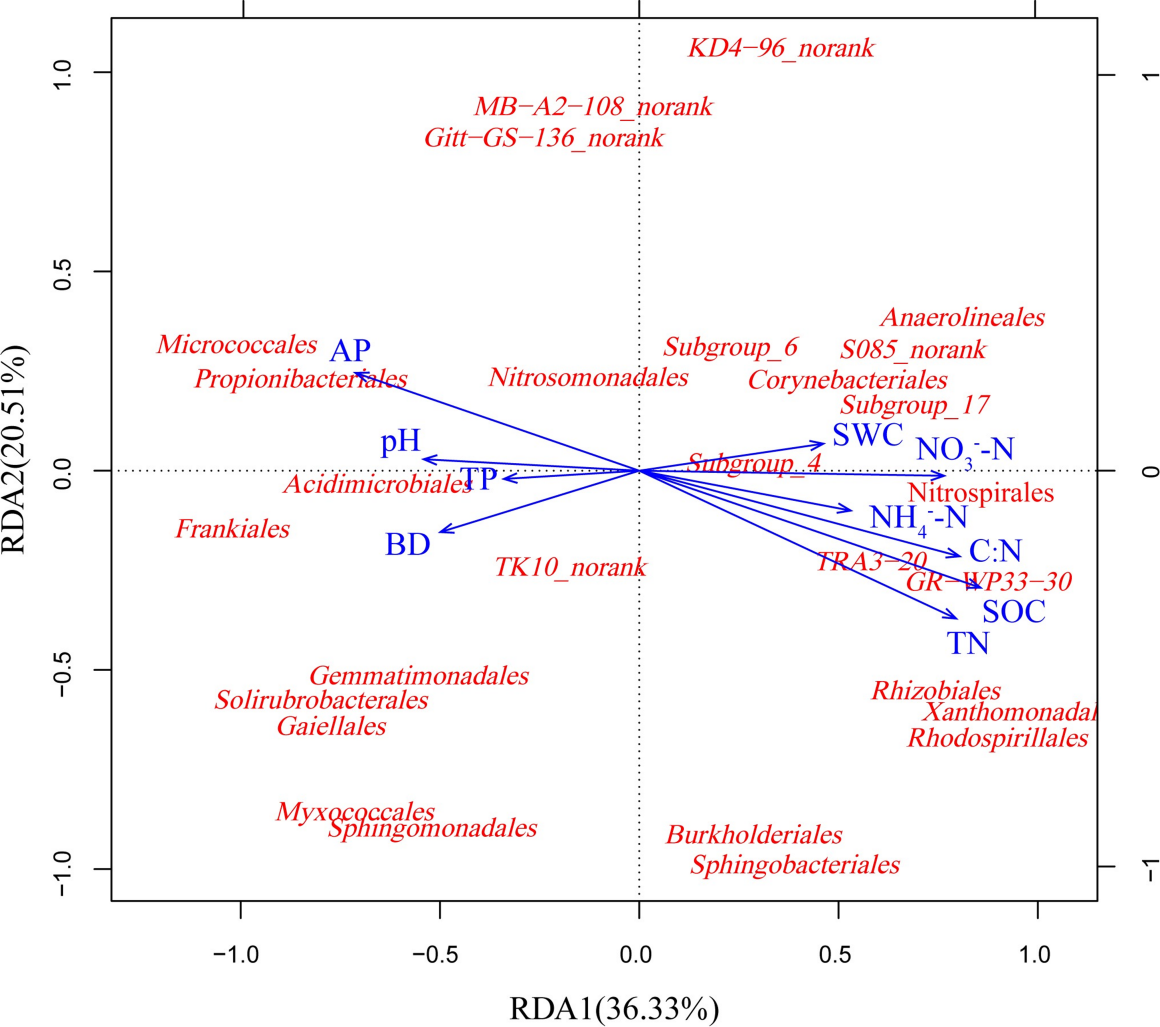
RDA of abundant bacterial communities at the order level and soil chemical properties for soil samples from abandoned farmland and Chinese pine forests.

**Fig 7:**
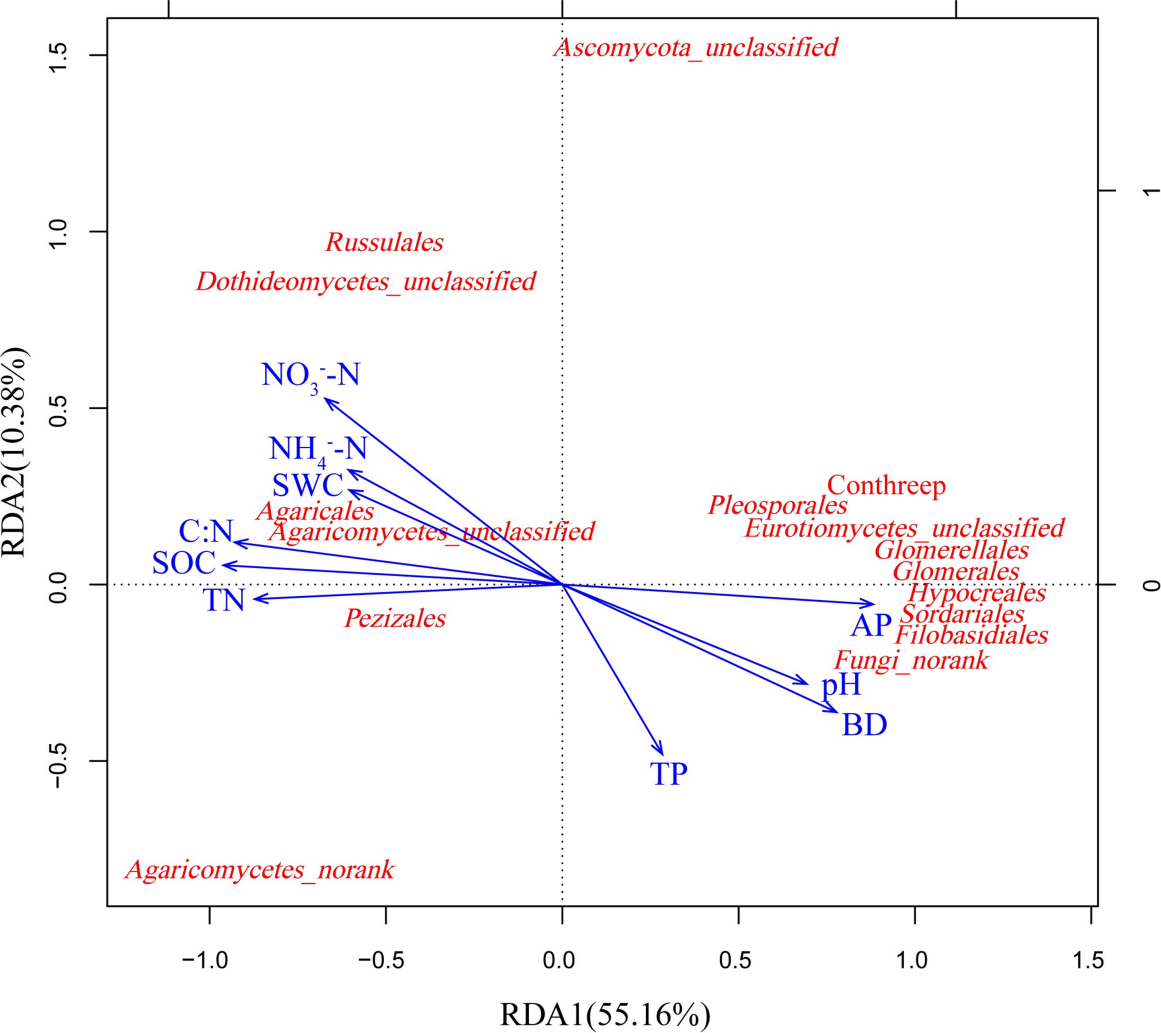
RDA of the dominant fungal communities at the order level and soil chemical properties for soil samples from abandoned land and Chinese pine forests.

The abundances of *Delta-* and *Gamma-proteobacteria*, *Bacteroidetes*, *Planctomycetes*, and *Basidiomycota* were correlated positively with the TOC, TN and C:N. In contrast, abundances of *Actinobacteria* and *Ascomycota* were correlated negatively with TOC, TN, and C: N. The abundances of *Actinobacteria*, *Ascomycota* and *Glomeromycota* were correlated positively with AP, while the abundances of *Nitrospirae*, *Planctomycetes* and *Basidiomycota* were correlated negatively with AP. The abundances of *Delta-* and *Gamma-proteobacteria* and *Nitrospirae* were correlated positively with NO_3_^-^-N (S1 Table.). The first two axes explained 56.84% and 65.54% of the total variance for the bacterial and fungal communities, respectively. Based on these results, SOC, TN, C:N, AP and NO_3_^-^-N showed close correlations with changes in the bacterial and fungal community composition (Figs 6 and 7). Soil bulk density (BD) was correlated negatively with fungal communities (Fig 7).

## Discussion

### Changes in microbial community diversity

The Shannon and Simpson indexes were determined to reveal differences in the complexity of soil microbial communities during the development of plantation stages. In our study, soil bacterial and fungal diversity was higher in PF1 than that in PF2, PF3 and NSF. However, the differences among PF2, PF3 and NSF were not significant. The soil microbial diversity of Chinese pine varied greatly among stands that were 12 and 29 years old during development of. However, after 29 years of age, the soil microbial diversity changed slightly. Plantation development affects soil microbes through several pathways [42–45]. For example, plantation trees cover directly affects the amount of light available and also affects the understory composition, which influences carbon allocation, the soil microbial community, nutrient cycling and water conservation of the soil. Hence, large differences in the plantation tree canopy between 12-year-old stands and other older stands may indirectly affect soil microbial diversity through the understory plants root system and litter substrates. Meanwhile, the major groups of *Actinobacteria* and *Ascomycota* demonstrated sharp decreases from the youngest 12-year-old plantation stands to the older stands undergoing succession. For example, most order-level fungal communities belonging to the *Ascomycota* phylum, significantly decreased after the 12-year-old plantation stage (S5 Fig). The decreases in these major groups, which were affected by soil nutrient and litter input, may contribute to the decline in microbial diversity observed with age in the study.

Clear differentiation in the PCoA plots illustrated that the bacterial and fungal communities at the BL and PF1 sites were similar to each other. Plantation stands in the later development stage (PF3) and NSF were similar to each other (Fig 3). The results indicate that afforestation plays an essential role in shaping microbial communities. With decades of plantation development, the soil microbial community structure of Chinese pine plantations has become similar to that of the NSF.

### Changes in the composition of bacterial and fungal communities

The relative abundance of bacterial phyla in the present study was similar to that of other soil environments reported in previous studies [16, 46]. In our study, the relative abundance of *Proteobacteria*, in forest soils was significantly greater than that in BL and increased with forest age. Similar to the previous finding [47], *Rhizobiales* was the most abundant order of *Proteobacteria* among all sample sites in our study. *Rhizobiales*, which fixes nitrogen from the atmosphere, increased with forest age in our study site. Thus, N-cycling groups of bacteria (e.g., *Rhizobiales*) play vital roles in the process of soil restoration in arid regions [48]. As one of the major bacterial groups, the *Actinobacteria* play important roles in organic matter turnover and carbon cycling [49]. The negative association between *Actinobacteria* and *Proteobacteria* abundance in our study sites is similar to previous studies in grassland soils [50] and snowmelt in an alpine tundra soil when plant carbon inputs were greatest [51]. It is assumed that the *Actinobacteria*, compared with the less abundant *Proteobacteria* group, are spore-forming bacteria, meaning that they dominate under harsh and stressful soil conditions [52].

*Basidiomycota* and *Ascomycota*, which represent the main soil fungi decomposers [53, 54], were the two major fungal communities, accounting for more than 90% of total fungal phyla. The *Agaricomycetes* (*Agaricales*, *Russulales*), belonging to the phylum *Basidiomycota*, contain ectomycorrhizal fungi related to pine development. *Agaricomycetes* increased substantially after afforestation. The most common orders of mycorrhizal fungi, such as *Russulales* and *Agaricales*, are late-stage fungi that are always symbiotic with older and larger trees [55, 56]. This association may be related to the storage of lignocellulose organic matter in older forests, which is degraded by members of *Basidiomycota* [57–59]. Moreover, large overstory trees contribute to the construction of a sustainable environment for mycorrhizal fungi [60, 61]. This may explain why mycorrhizal fungi occupied a dominant position in the Chinese pine plantation stands 29-year and older in our study. Mycorrhizal fungi play important roles in the pine forest ecosystem by promoting the absorption and utilization of mineral elements in the roots of trees. After approximately 29 years of afforestation recovery, the Chinese pine plantation gradually formed a stable ecosystem, which provides a sustainable habitat environment for mycorrhizal fungi. However, the relative abundances of *Ascomycota* were greater in BL and PF1 than those in the other sites. Abundance was similar among the PF2, PF3 and NSF sites. Apparently, *Ascomycota* gradually withdraw from a dominant advantage when Chinese pine plantation growth progresses to later stages. We propose that shifts in the fungal community structure are mediated by the tree structure and plant cover after afforestation with Chinese pine trees, which is consistent with previous studies [62, 63].

### Response of the microbial community to soil physicochemical properties

Previous studies showed that SOC played the most important role in microbial community structure, and N availability also had an impact on soil microbial communities [50, 64]. In our study, the relative abundances of *Proteobacteria*, *Bacteroidetes* and *Nitrospirae* were correlated positively with the SOC and TN concentrations. Similar results have been reported previously [65–67]. This finding indicates that these bacteria prefer enriched soil environments and belong to eutrophic groups. This finding supports previous reports that abundance of *Proteobacteria* and *Bacteroidetes* represent soils with abundant labile substrates, exhibiting copiotrophic attributes [16, 66]. As the dominant bacterial group of this study, *Proteobacteria* played a comparable functional role in long term plantation restoration in arid and semi-arid lands.

However, the relative abundance of *Actinobacteria* was correlated negatively with the SOC, TN, and C:N, similar to previous studies that *Actinobacteria* was linked with low carbon concentrations in temperate [49, 68, 69] and Arctic [70] soils with one exception [71]. In the exception [71], the abundance of *Actinobacteria* increased with nitrogen inputs, and suggested that the *Actinobacteria* may represent a copiotrophic group. Whether *Actinobacteria* populations belong to copiotrophic or oligotrophic groups remains an open question [65]. *Actinobacteria* may adopt an oligotrophic life strategy in the study sites environment. Meanwhile, abundance of *Actinobacteria* decreased markedly when croplands were converted to monoculture plantation [49]. In this latter case, the abundance of *Actinobacteria* correlated positively with the SOC mineralization rate. This study indicates that the conversion of cropland to forests decreases the amount of *Actinobacteria* abundance that are capable of decomposing more recalcitrant soil carbon. *Actinobacteria* may be well adapted to harsh environmental conditioning due to its metabolic versatility and widespread occurrence in pristine soil [72, 73].

Previous studies showed that NO_3_^-^-N was a main soil-available element influenced by bacterial communities on the Tibetan Plateau [74]. Additionally, nitrogen addition and soil NO_3_^-^-N content positively influenced the relative abundances of dominant bacterial groups [75, 76]. Zhang et al. [16] showed that NO_3_^-^-N closely correlated with the relative abundances of bacterial communities during the natural succession of abandoned land on the loess plateau. Similarly, this result indicated that NO_3_^-^-N was a dominant chemical factor that influences soil bacterial communities during plantation ecological processes on the Loess plateau. Thus, these dominant bacterial communities may be regulated by soil nutrients, particularly the soil carbon and nitrogen.

*Basidiomycota* were positively correlated with TOC, TN, C:N, and NO_3_^-^-N. Values of TOC, TN and C:N ratio increased gradually with forest age. *Basidiomycota* play important roles in mediating the decomposition of low-quality lignified and aromatic substrates, which accumulate with increases in the tree cover and understory vegetation diversity of both coniferous and deciduous tree litter [77–79]. This finding explains why pine forest soils and hardwoods with high C:N ratios demonstrate a high prevalence of *Basidiomycetes*. The abundance of *Ascomycota* showed a positive correlation with AP. Meanwhile, Previous studies also considered the relationship between the relative abundance of *Ascomycota* and soil phosphorous content in soils. Therefore, Soil phosphorous is considered an important regulator of fungal communities in the soil [80].

## Conclusion

Our study suggests that plantations influence soil properties and microbial communities. Microbial communities correlated with soil factors during the process of vegetation restoration. Obvious decreases in bacterial and fungal community diversity indexes occurred between 12 and 29 years after plantation. The relative abundance of *Proteobacteria* increased with age over 53 years of growth. However, the relative abundance of *Actinobacteria* decreased between 12 years and 29 years after plantation. Fungal communities transitioned from being *Ascomycota* dominant in the BL and 12-year-old plantation stand sites to *Basidiomycota* dominant after 29 years. These bacterial and fungal phyla reflected the patterns of belowground microorganisms during the process of plantation restoration in the Loess Plateau. Soil microbial community composition and diversity reached relatively stable states after 29 years of growth. Change in microbial communities correlated with changes in the availability of soil nutrients (SOC, TN, AP, C:N, and NO_3_^-^-N).

## Supporting information

S1 Fig Rarefaction curves for soil bacterial (a) and fungal (b) communities at 97% sequence similarity in soil.
(TIF)

S2 Fig Mean relative abundances of *Proteobacteria* populations at the class level at each site. (TIF)

S3 Fig Mean relative abundances of *Proteobacteria* populations at the order level at each site. (TIF)

S4 Fig Mean relative abundances of *Actinobacteria* populations at the order level in each site. (TIF)

S5 Fig Mean relative abundances of dominant fungal orders in each site. (TIF)

S1 Table Correlations among dominant bacterial and fungal communities (phyla) and soil properties. (PDF)
