# Supplementary figures and images for: Effects of stand age and soil properties on soil bacterial and fungal community composition in Chinese pine plantations on the Loess Plateau
Source: PLoS One. 2017 Oct 19;12(10):e0186501. doi: 10.1371/journal.pone.0186501 (PMC5648195; doi:10.1371/journal.pone.0186501)

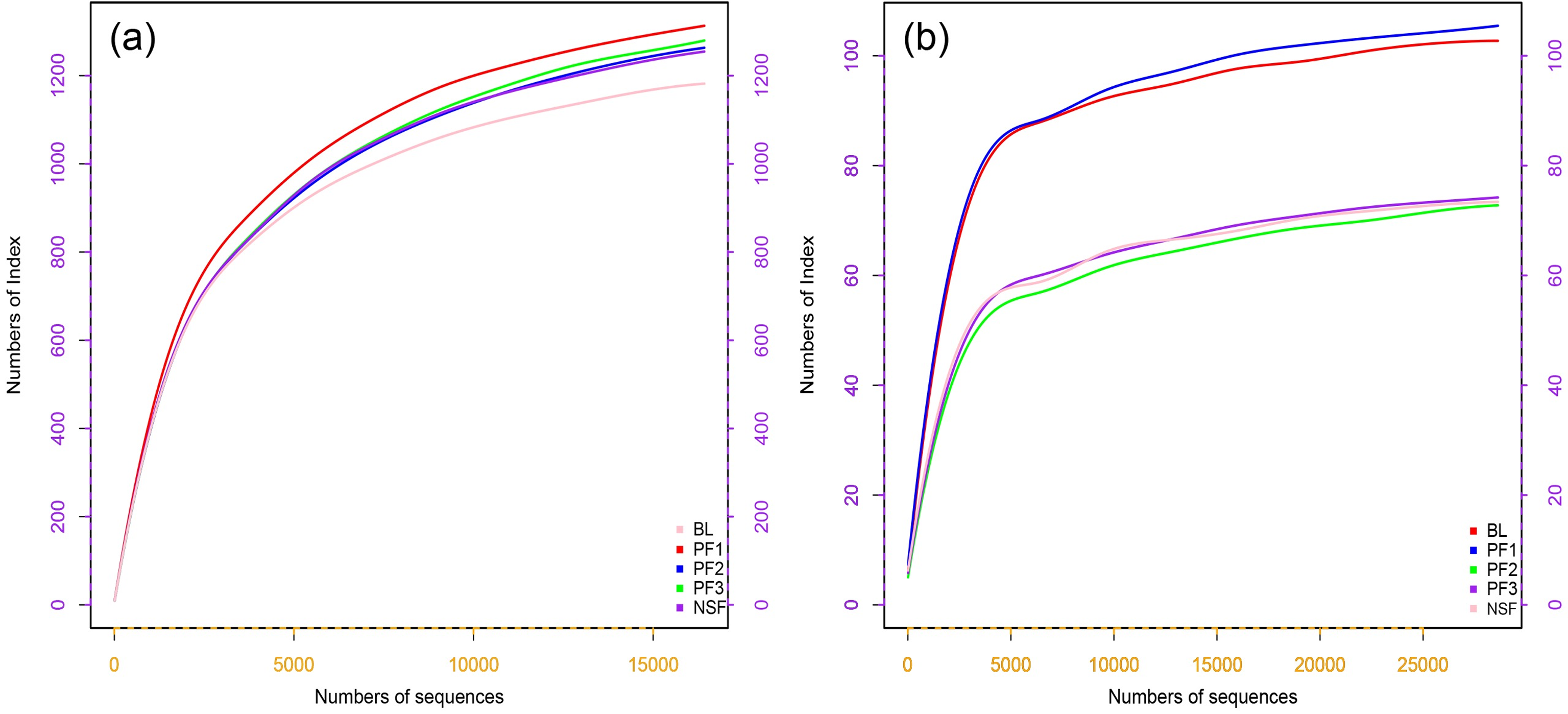

Supplement: S1 Fig — Rarefaction curves for soil bacterial (a) and fungal (b) communities at 97% sequence similarity in soil. (TIF) [file pone.0186501.s001.tif]

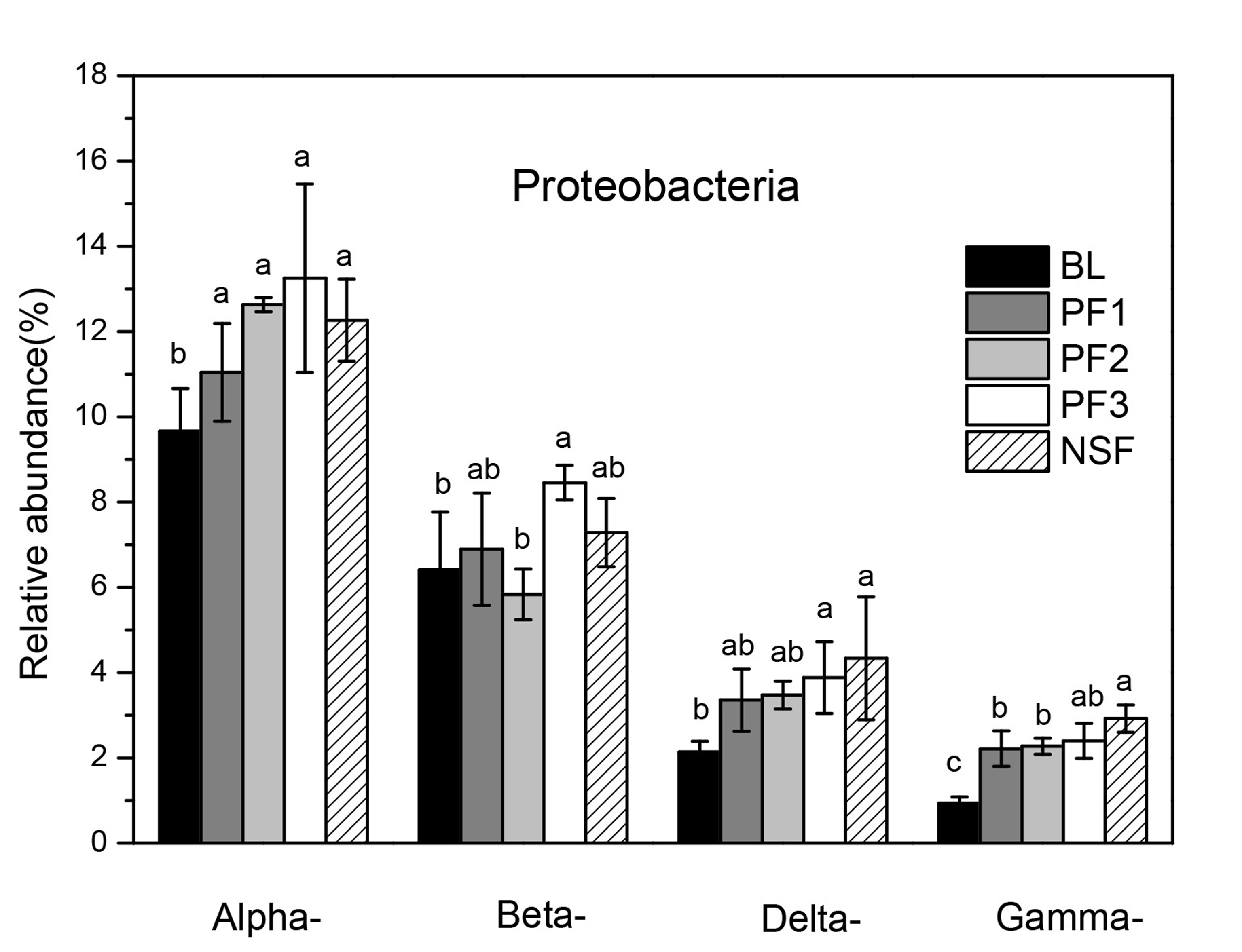

Supplement: S2 Fig — (TIF) [file pone.0186501.s002.tif]

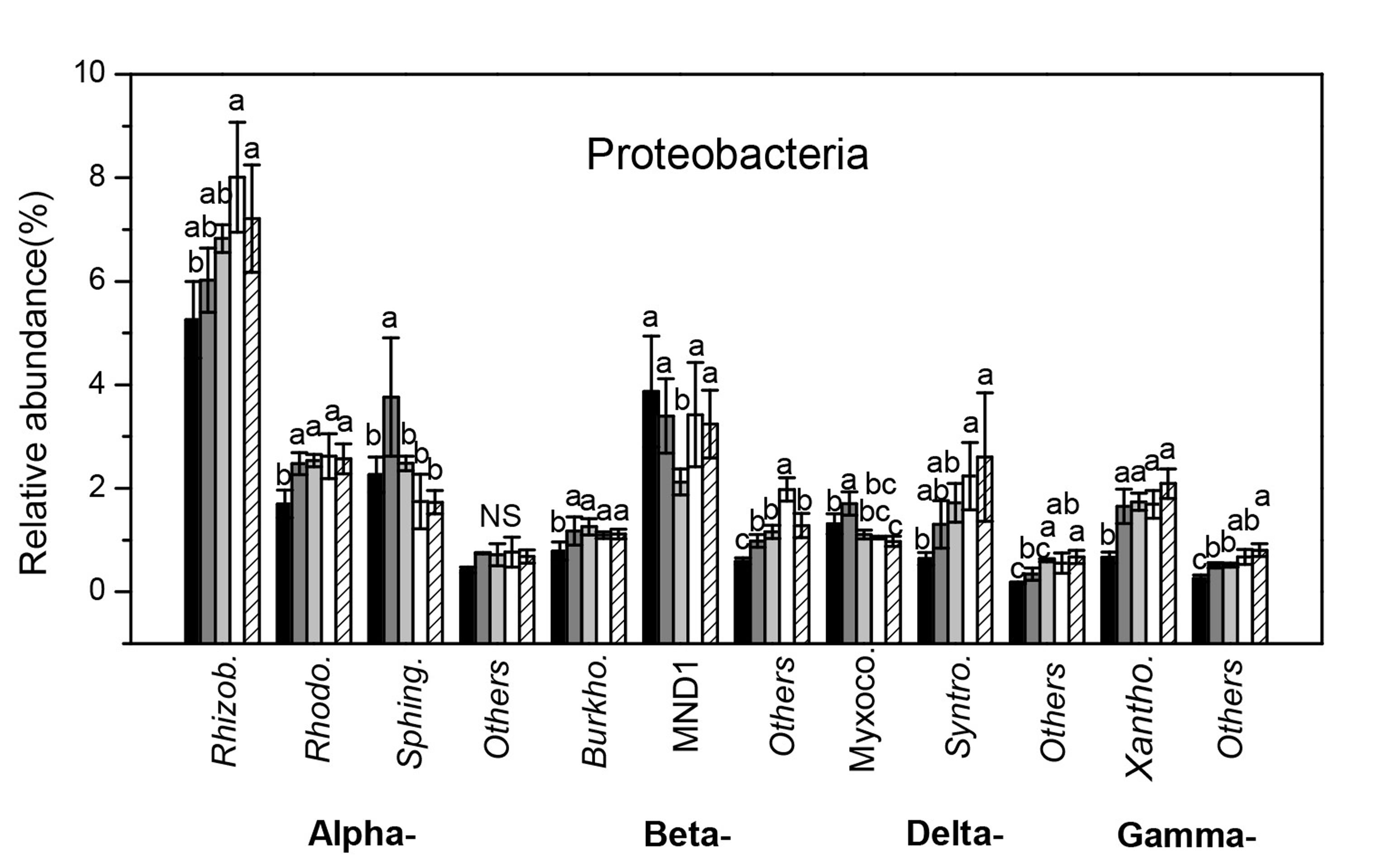

Supplement: S3 Fig — (TIF) [file pone.0186501.s003.tif]

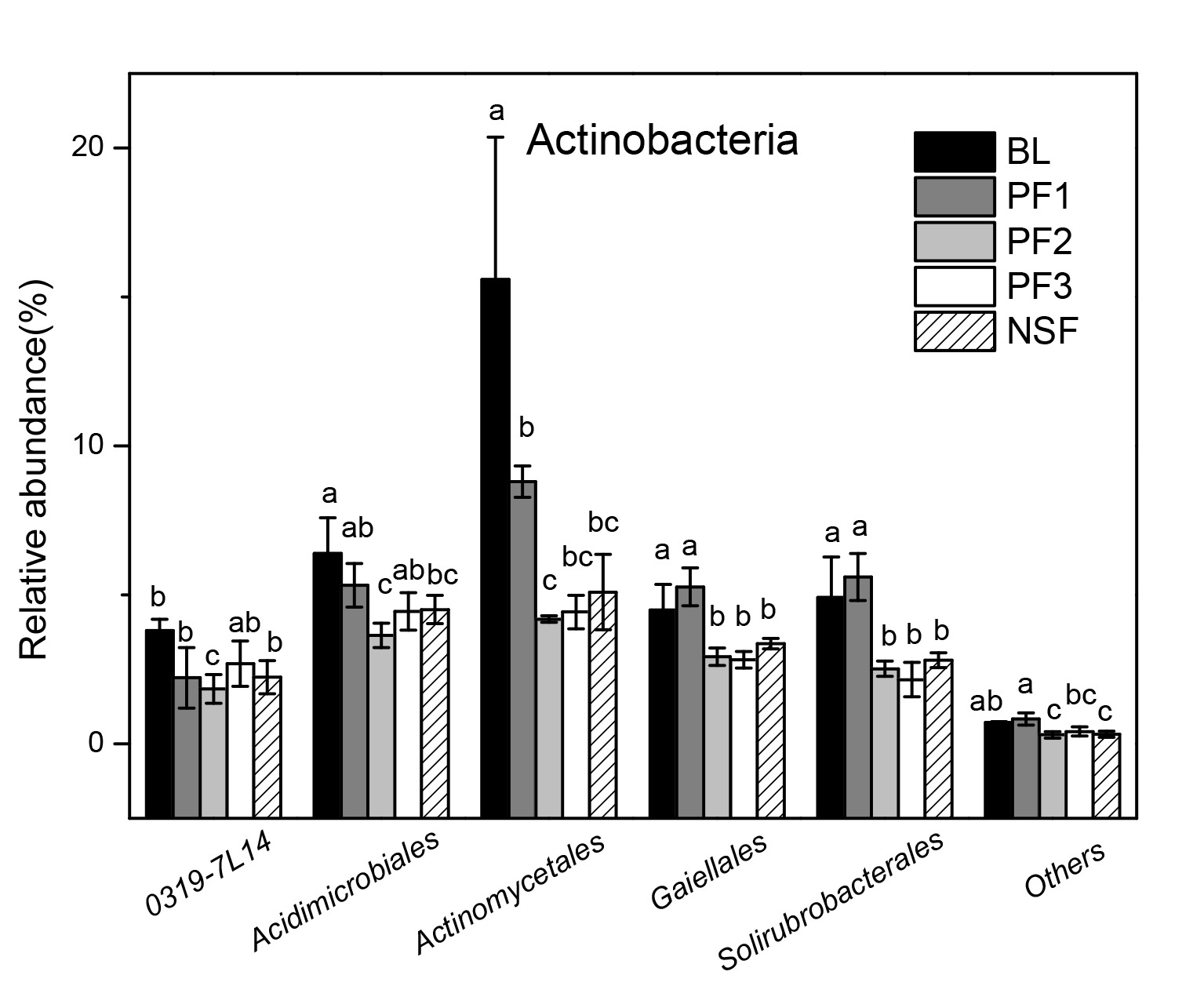

Supplement: S4 Fig — (TIF) [file pone.0186501.s004.tif]

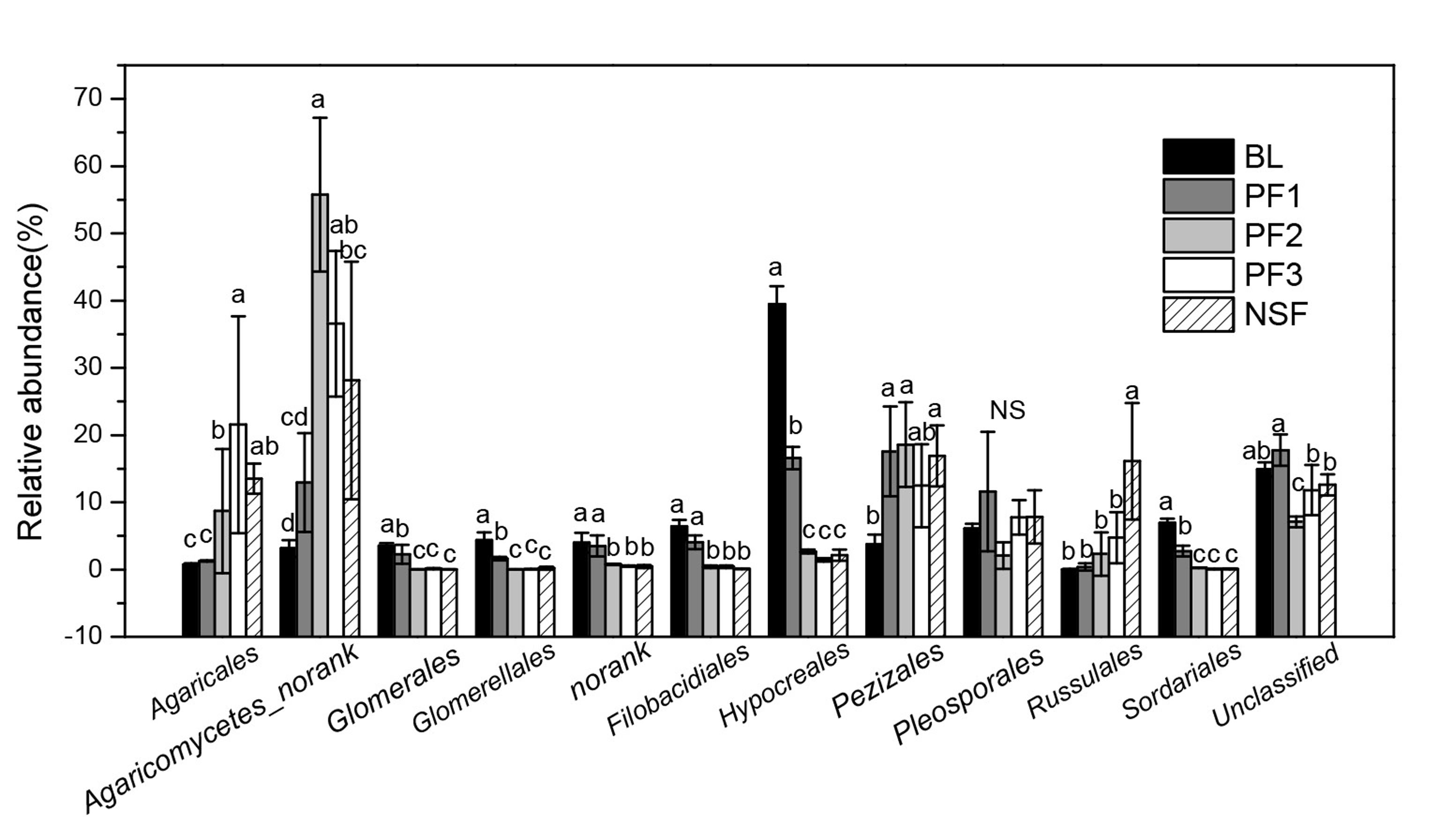

Supplement: S5 Fig — (TIF) [file pone.0186501.s005.tif]
