# Supplementary material for: Effects of stand age and soil properties on soil bacterial and fungal community composition in Chinese pine plantations on the Loess Plateau
Source: PLoS One. 2017 Oct 19;12(10):e0186501. doi: 10.1371/journal.pone.0186501 (PMC5648195; doi:10.1371/journal.pone.0186501)
Supplement: S1 Table — (PDF) [file pone.0186501.s006.pdf]

Table S1 Correlations among dominant bacterial and fungal communities (phyla) and soil properties.

	pH	SOC (g/kg)	TN (g/kg)	TP (g/kg)	C: N	NH_4^--N (mg/kg)	NO_3^--N (mg/kg)	AP (mg/kg)	SWC (%)	BD (g/cm^3)
Proteobacteria	-0.534*	0.726**	0.681**	-0.545*	0.697**	0.527*	0.647**	-0.636*	0.587*	-0.504*
Actinobacteria	0.472	-0.796**	-0.757**	0.167	-0.746**	-0.405	-0.5	0.667**	-0.44	0.317
Acidobacteria	0.131	0.241	0.37	0.465	0.102	-0.127	-0.238	-0.118	-0.244	0.196
Chloroflexi	-0.205	-0.09	-0.273	-0.195	0.065	0.058	0.297	0.043	0.312	-0.388
Nitrospirae	-0.572*	0.611*	0.489	-0.61*	0.637*	0.505	0.734**	-0.577*	0.545*	-0.594*
Gemmatimonadetes	0.194	-0.293	-0.391	-0.077	-0.145	0.05	-0.125	0.165	0.023	0.221
Bacteroidetes	-0.299	0.639*	0.647**	-0.114	0.585*	0.274	0.244	-0.545*	0.335	-0.269
Alphaproteobacteria	-0.229	0.552*	0.629*	-0.26	0.445	0.331	0.317	-0.44	0.358	-0.344
Betaproteobacteria	-0.504	0.283	0.119	-0.628*	0.408	0.264	0.521*	-0.342	0.633*	-0.549*
Deltaproteobacteria	-0.548*	0.715**	0.655**	-0.531*	0.678**	0.525*	0.693**	-0.615*	0.453	-0.528*
Gammaproteobacteria	-0.622*	0.886**	0.786**	-0.409	0.871**	0.738**	0.752**	-0.759**	0.473	-0.640*
Ascomycota	0.570*	-0.767**	-0.689**	0.198	-0.744**	-0.427	-0.456	0.667**	-0.431	0.602*
Basidiomycota	-0.590*	0.788**	0.710**	-0.212	0.763**	0.441	0.479	-0.685**	0.45	-0.618*
Fungi_norank	0.683**	-0.751**	-0.707**	0.264	-0.702**	-0.36	-0.563*	0.690**	-0.505	0.605*
Glomeromycota	0.550*	-0.854**	-0.763**	0.303	-0.835**	-0.605*	-0.567*	0.693**	-0.495	0.659**
Mucoromycotina	-0.244	0.159	0.166	-0.538*	0.116	0.39	0.583*	-0.056	0.189	-0.378
Chytridiomycota	0.683**	-0.807**	-0.795**	0.24	-0.74**	-0.421	-0.493	0.800**	-0.489	0.522*
Zoopagales	0.459	-0.586*	-0.508	0.145	-0.587*	-0.222	-0.334	0.427	-0.345	0.367

**Significant at $P<0.01$, * Significant at $P<0.05$;

SOC, soil organic carbon; TN, soil total nitrogen; TP, soil total phosphorus; C: N, soil organic carbon and total nitrogen ratio; NH_4^+-N, ammonium nitrogen; NO_3^--N, nitrate nitrogen; AP, available phosphorus, SWC, soil water content; BD, soil bulk density.
